# Vitamin D in Atopic Dermatitis: Role in Disease and Skin Microbiome

**DOI:** 10.3390/nu17223584

**Published:** 2025-11-16

**Authors:** Karolina Blady, Bartosz Pomianowski, Miłosz Strugała, Leon Smółka, Karolina Kursa, Agata Stanek

**Affiliations:** 1 Student Scientific Association, Department of Internal Medicine, Metabolic Diseases, and Angiology, Faculty of Health Sciences, Medical University of Silesia, Ziolowa 45/47 St., 40-635 Katowice, Poland; s83086@365.sum.edu.pl (B.P.); s83152@365.sum.edu.pl (M.S.); s81350@365.sum.edu.pl (L.S.); s91506@365.sum.edu.pl (K.K.); 2 Department of Internal Medicine, Metabolic Diseases, and Angiology, Faculty of Health Sciences in Katowice, Medical University of Silesia, Ziolowa 45/47 St., 40-635 Katowice, Poland; 3 Upper-Silesian Medical Center, 45/47 Ziołowa St., 40-635 Katowice, Poland

**Keywords:** nutrition, microbiome, vitamin D, mercury, skin diseases, atopic dermatitis, inflammation

## Abstract

Atopic dermatitis (AD) is a chronic inflammatory skin disorder associated with immune dysregulation, skin barrier dysfunction, and microbial dysbiosis characterized by *Staphylococcus aureus* overcolonization and reduced bacterial diversity. Beyond its classical role in calcium homeostasis, Vitamin D (VD) influences skin immunity and microbial composition. This review summarizes current knowledge on VD metabolism, its immunological pathways in AD, and its interactions with the skin microbiome. Recent evidence positions the skin as an active immunological organ rather than a passive barrier. Commensal bacteria such as *Staphylococcus epidermidis* not only inhibit pathogens by producing bacteriocins and modulins but also generate ceramides and short-chain fatty acids (SCFAs) that stabilize the lipid barrier. Moreover, dermal fibroblasts and preadipocytes produce antimicrobial peptides, while resident γδ T cells release growth factors like fibroblast growth factor 7 (FGF7), linking host defense with tissue regeneration. VD modulates AD by suppressing T helper 2 cells/T helper 17 cell responses, enhancing regulatory T cell development, inducing antimicrobial peptides, and strengthening skin and gut barrier integrity. Its interaction with the microbiome and pathways such as SCFA and aryl hydrocarbon receptor (AhR) signaling supports its potential as an adjunctive therapy in AD management. Evidence from mechanistic studies and animal models suggests that VD supplementation may modulate inflammation and microbial diversity. Clinical implications, therapeutic perspectives, and future research directions highlight the potential of VD as a therapeutic adjunct in AD management.

## 1. Introduction

Atopic dermatitis (AD) is a multifactorial chronic disease characterized by impaired epidermal barrier function [1,2]. It manifests as recurrent inflammatory skin lesions accompanied by pruritus [3], which significantly reduce patients’ quality of life, particularly in moderate-to-severe forms of the disease. Approximately 50% of adult patients with AD report experiencing social exclusion [4]. Sleep disturbances are common, and additional burdens include time-consuming skin care routines and dietary modifications [5,6]. Although some patients experience long periods of remission, in others, the disease presents as persistently active chronic skin inflammation [7]. The pathogenesis of AD is multifactorial, involving epidermal barrier dysfunction, dysregulated immune responses, and alterations in the skin microbiome [8]. A hallmark of allergic diseases, including AD, is elevated serum immunoglobulin E (IgE) levels [9]. Patients show enhanced type 2 immune responses, with increased production of interleukin- 4 (IL-4), IL-5, IL-13, and IL-31, along with reduced expression of filaggrin and antimicrobial peptides (AMPs) in the skin [10,11,12]. Cluster of differentiation 4 positive (CD4+) T helper type 2 (Th2) cells play a central role in this process, as excessive activation of these cells amplifies the inflammatory cascade typical of AD [13,14]. Cytokines produced by Th22 and Th17 cells may also contribute to disease pathogenesis in certain patients [15]. A healthy skin microbiome is essential for maintaining cutaneous homeostasis, but in AD, microbial diversity is markedly reduced [16,17]. Up to 70% of patients exhibit increased colonization by *Staphylococcus aureus*, which correlates with disease severity [18,19]. In adults with AD, changes in the skin microbiome include not only the dominance of *Staphylococcus aureus* but also a significant decrease in microbial diversity, which correlates with the severity of clinical symptoms assessed using the Scoring Atopic Dermatitis (SCORAD) and Eczema Area and Severity Index (EASI) scales [20,21]. The role of *Staphylococcus epidermidis* appears to be phase-dependent: during remission, it supports microbial balance and suppresses *Staphylococcus aureus*, whereas during flares, it may be outcompeted or represented by pro-inflammatory strains [21,22].

Genetic studies have revealed that both well-known genes such as filaggrin (FLG) and rare variants affecting signaling pathways (e.g., signal transducer and activator of transcription 6 (STAT6), janus kinase 1 (JAK1)) are implicated in AD pathogenesis [23]. According to one hypothesis, FLG gene variants may have provided an adaptive function by facilitating vitamin D (VD) synthesis in populations living in regions with low sunlight exposure [24].

The skin of patients with AD typically demonstrates increased pH, greater permeability, and reduced intercellular cohesion, all of which promote bacterial overgrowth and facilitate penetration of irritants [25]. Symptom exacerbation is frequently observed during winter months in regions with limited sun exposure, when cutaneous VD synthesis is at its lowest [26,27]. Indeed, reduced serum VD concentrations have been documented in patients with AD, particularly in those with moderate to severe disease [28,29]. The skin expresses the vitamin D receptor (VDR), the activation of which is pivotal for regulating the production of human cathelicidin antimicrobial peptide (LL-37), an antimicrobial peptide integral to innate host defense [30]. Moreover, VD metabolism within keratinocytes promotes their proliferation and differentiation, thereby contributing to barrier repair and maintenance of epidermal integrity [31,32]. Genetic polymorphisms in the VDR gene have been associated with susceptibility to AD [33,34,35]. In recent years, studies have shown that VD supplementation may beneficially modulate the microbiome, reducing AD symptom severity [36,37].

The aim of this review is to analyze the impact of VD supplementation on skin microbiome modulation in patients with AD.

AD is a chronic inflammatory skin disorder characterized by a complex confluence of genetic predisposition, epidermal barrier dysfunction, immune dysregulation, and microbial imbalance. Mutations in the FLG gene and decreased levels of ceramides and antimicrobial peptides impair the skin’s barrier integrity, facilitating allergen penetration and microbial colonization, particularly by *Staphylococcus aureus*.

This barrier impairment triggers a predominant Th2-type immune response with elevated IL-4, IL-13, and IgE levels, while reduced microbial diversity further amplifies inflammation. Together, these mechanisms establish a vicious cycle linking the barrier, immune system, and microbiome in AD pathogenesis.

## 2. Materials and Methods

For this review, a systematic search for scientific publications on the role of VD in AD and its effects on both the skin and gut microbiome was conducted in the PubMed, Scopus, and Web of Science databases, covering studies published until September 2025.

A combination of the following keywords was used: “vitamin D”, “atopic dermatitis”, “skin microbiome”, “gut-skin axis”, “VDR”, and “Th17/Treg balance”. Original articles, review papers, meta-analyses, and both randomized and observational clinical studies published in English were included.

**Figure 1:**
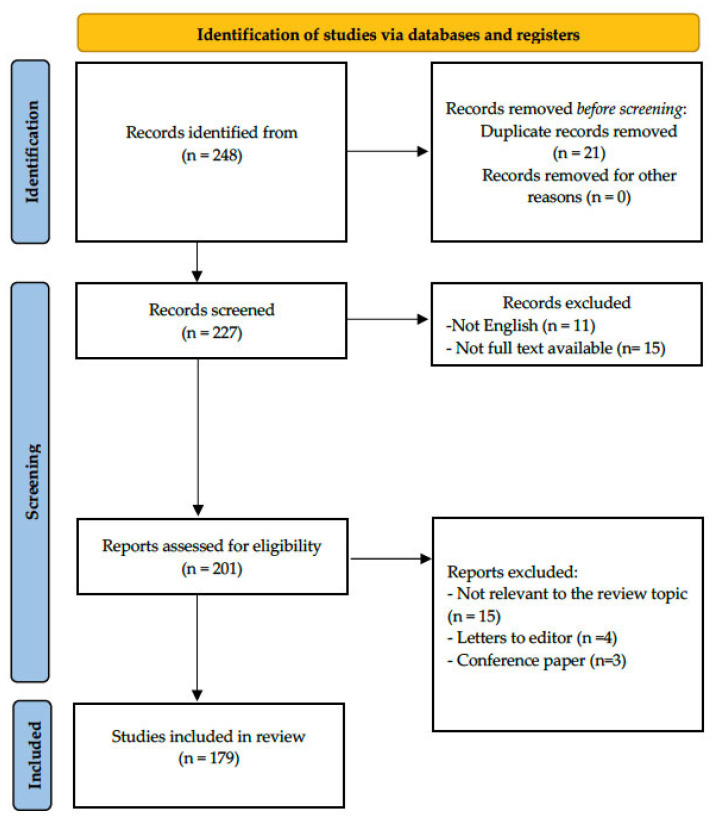
PRISMA flow diagram for study selection.

The analysis focused on studies involving patients with AD, as well as animal and cell models providing mechanistic insights. Non- full-text papers, conference abstracts, and studies unrelated to the topic were excluded (Figure 1).

The selected data were categorized according to thematic domains: immunomodulation, barrier function, microbiome alterations, and molecular mechanisms.

## 3. Vitamin D Metabolism and Skin Immune Mechanisms

### 3.1. Vitamin D Metabolism

**Figure 2:**
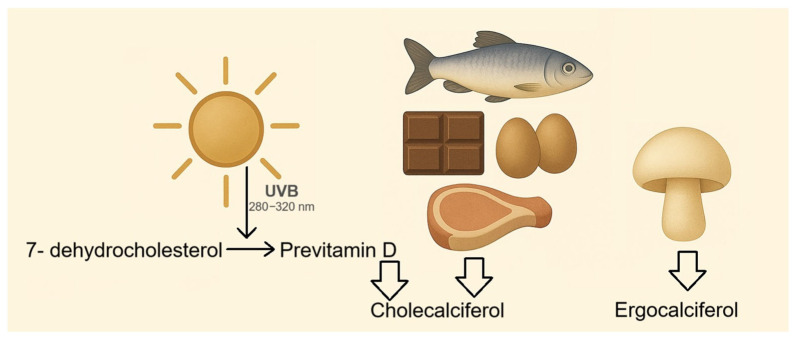
Synthesis and dietary sources of vitamin D. Under UVB (280–320 nm), 7-dehydrocholesterol in the skin is converted into previtamin D_3_ and then into cholecalciferol (VD3). VD3 is also obtained from animal-based foods such as fish, eggs, meat and chocolate, while ergocalciferol (VD2) originates from plant sources, mainly mushrooms.

VD refers to a group of fat-soluble steroid compounds that play a pivotal role in calcium homeostasis and the proper functioning of the immune system [38]. Although VD can be obtained from dietary sources, such as fish, meat, eggs, chocolate, and mushrooms (Figure 2) [39], achieving the recommended daily intake (600–800 IU/day) through diet alone is difficult. VD deficiency is common worldwide, particularly among populations living in temperate and polar regions, where annual sunlight exposure is limited. It is estimated that approximately 40% of the European population is VD deficient [40]. Thus, cutaneous synthesis under sunlight exposure remains the primary source for meeting daily requirements. Studies indicate that 35 min of daily sun exposure is sufficient to generate about 1000 IU of VD, which is adequate to maintain appropriate serum concentrations [41].

**Figure 3:**
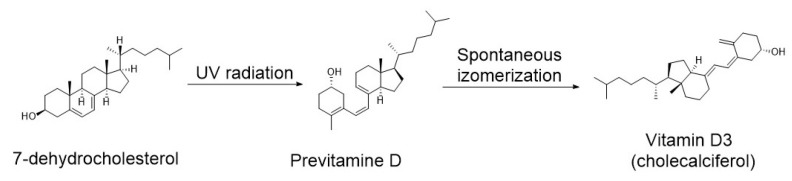
Conversion of 7-dehydrocholesterolaryl (7-DHC) to previtamin D under UVB and the spontaneous isomerization of previtamin D to cholecalciferol (VD3).

VD is obtained from the diet in two forms: ergocalciferol (VD2), which is derived from plant-based foods and fungi, and cholecalciferol (VD3), which is derived from animal-based products. Both forms are biologically inactive and require enzymatic conversion to their active metabolites. However, approximately 90% of the body’s VD pool originates from endogenous cutaneous synthesis, with only about 10% obtained from dietary sources [41], underscoring the importance of regular sunlight exposure. Cholecalciferol is synthesized in the skin upon exposure to ultraviolet B radiation (UVB) with wavelengths between 280 and 320 nm. This radiation induces the conversion of 7-dehydrocholesterol (7-DHC), which is present in the stratum spinosum and stratum granulosum of the epidermis, into cholecalciferol (Figure 1). When sunlight containing UVB reaches uncovered skin, it triggers the opening of the B-ring in the 7-DHC molecule, forming an intermediate compound known as previtamin D_3_. This compound then undergoes spontaneous thermal isomerization to produce VD3 (Figure 3) [42].

**Figure 4:**
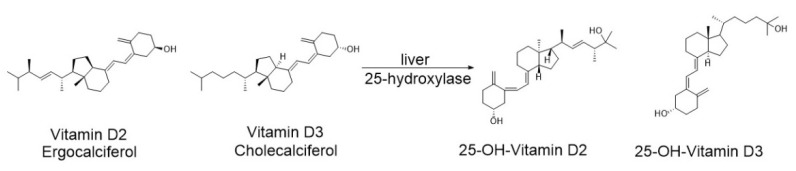
Enzymatic hydroxylation of ergocalciferol (VD2) and cholecalciferol (VD3) to 25-hydroxycholecalciferol and 25-hydroxyergocalciferol in the liver.

Dietary forms of VD are absorbed in the small intestine by enterocytes through passive diffusion, as well as with the assistance of cholesterol transport proteins such as Niemann-Pick C1-like protein 1 (NPC1L1), scavenger receptor class B type I (SR-BI), and CD36 [43,44]. Following absorption, they are transported into the lymphatic system and subsequently into the bloodstream. In circulation, they are predominantly bound to Vitamin D-binding protein (DBP); although to a lesser extent, they are also carried by albumin or present in a free form. Within the liver, VD undergoes enzymatic hydroxylation and bioactivation to 25-(OH)D_3_ (25-hydroxycholecalciferol, calcifediol) and 25-(OH)D_2_ (25-hydroxyergocalciferol, calcidiol) (Figure 4) [45], mediated by the enzyme 25-hydroxylase encoded by the CYP2R1 gene. This product serves as the primary indicator of VD status in the body and is routinely measured in clinical practice to assess serum VD levels. However, it exhibits only weak biological activity.

**Figure 5:**
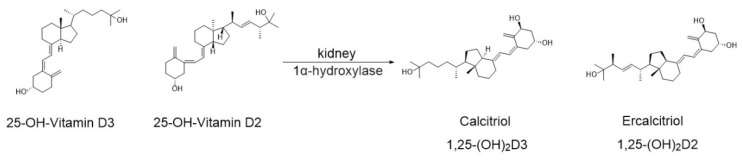
Conversion of calcifediol and calcidiol to calcitriol (1,25(OH)_2_D_3_) and ercalcitriol (1,25(OH)_2_D_2_) in the kidneys under the action of α1-hydroxylase.

Subsequently, 25(OH)D bound to DBP and albumin is transported to the kidneys. There, under the action of the enzyme 1α-hydroxylase, calcifediol and calcidiol are converted into calcitriol (1,25-dihydroxycholecalciferol, 1,25(OH)_2_D_3_) and ercalcitriol (1,25-dihydroxyergocalciferol, 1,25(OH)_2_D_2_), respectively (Figure 5). Of these two metabolites, calcitriol exhibits the higher biological activity and is considered the most active form of VD [46].

**Figure 6:**
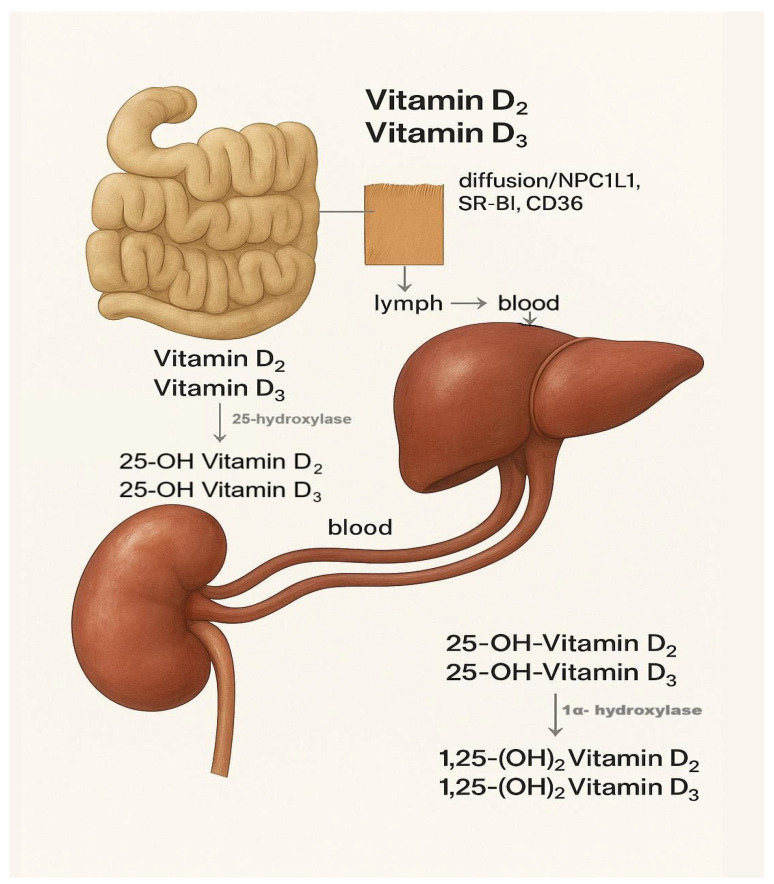
Transport and metabolism of vitamin D_2_ (ergocalciferol) and D_3_ (cholecalciferol). Vitamins D_2_ and D_3_ are absorbed in the intestine via passive diffusion and transporters (NPC1L1, SR-BI, and CD36) and enter the bloodstream through the lymphatic system. In the liver, they are hydroxylated by 25-hydroxylase to form 25-hydroxyvitamin D_2_ and D_3_. These metabolites are further hydroxylated in the kidneys by 1α-hydroxylase to produce the biologically active forms, 1,25-dihydroxyvitamin D_2_ and D_3_.

The active form of VD then enters target cells, where, by binding to nuclear VDR, a transcription factor, it regulates gene expression through transcriptional activation and transrepression of specific target genes [47] (Figure 6).

Mutations in genes encoding enzymes of the VD synthesis pathway are an important cause of functional VD deficiency, which may occur even in the presence of normal laboratory values. Mutations or deletions of the CYP2R1 gene are associated with VD-dependent rickets type 1b. Although this is a relatively rare disorder, defective alleles encoding 25-hydroxylase occur with a frequency of 3 in 1000 births in the Caucasian population [48]. Patients with VD-dependent rickets type 1b require supplementation with calcifediol and calcium [49]. Another clinically relevant gene is CYP27B1, which encodes 1α-hydroxylase, the enzyme responsible for the conversion of calcifediol and calcidiol to calcitriol and ercalcitriol. Mutations in CYP27B1 cause VD-dependent rickets type 1a (also referred to as pseudovitamin D deficiency rickets). Although biochemical testing may show normal or even elevated concentrations of 1,25(OH)_2_D_3_, the inability to generate its active form in the kidneys results in clinical manifestations of VD deficiency in early childhood [50]. Treatment involves supplementation with calcitriol and phosphate; early diagnosis enables good disease control and complete resolution of symptoms. In the case of mutations in the VDR gene, which encodes the nucVD receptor, patients develop hypocalcemic rickets resistant to VD. This disorder is characterized by reduced tissue responsiveness to VD, and one of its hallmark clinical features is near-total alopecia [51].

### 3.2. Skin Immune Mechanisms

The skin is the largest organ of the human body [52], serving as a key barrier that prevents microbial invasion. All anatomical layers of the skin contribute to antimicrobial defense through interlayer interactions or layer-specific mechanisms [53]. Breaches in skin integrity under non-sterile conditions may result in wound infections and, in severe cases, sepsis and death if adequate prophylactic antibiotic therapy is not initiated.

The skin consists of three layers: the outer epidermis, the dermis, and the subcutaneous tissue [54]. The epidermis is a stratified squamous keratinized epithelium in which keratinocytes are tightly interconnected by desmosomes, hemidesmosomes, tight junctions (TJs), and adherens junctions. These structures ensure impermeability to pathogens and maintain skin homeostasis [55]. The epidermis is colonized by a unique microbiome composed predominantly of commensal bacteria, viruses, and fungi, which vary between individuals [56]. These organisms primarily utilize lipids secreted by sebaceous glands located in the dermis, preventing their use by pathogenic bacteria attempting to colonize the skin. Common epidermal residents include *Staphylococcus epidermidis* and *Cutibacterium acnes*, which exert protective effects by limiting the growth of pathogenic bacteria such as *Staphylococcus aureus* and *Streptococcus pyogenes*, both of which are associated with skin disease [57].They help maintain an acidic pH, which makes the skin a harsh environment for many pathogens and supports its structural integrity [58]. Moreover, commensal bacteria play a crucial role in preventing excessive inflammation and helping regulate immune responses [59].

Commensal bacteria perform several antimicrobial functions, including the production of bacteriocins, ribosomally synthesized proteins that bind to receptors on the cell walls of susceptible species, inhibiting their growth or inducing cell death [60]. Another antimicrobial mechanism involves phenol-soluble modulins, amphipathic cytolytic molecules that act synergistically with bacteriocins. *Staphylococcus epidermidis*-derived modulins are active against *Staphylococcus aureus* and group *A streptococci* [61]. Commensal microbes also contribute maintaining the skin’s acidic pH by producing short-chain fatty acids (SCFAs), including butyric and propionic acids [62]. This acidic environment inhibits pathogen overgrowth while favoring beneficial residents such as *Cutibacterium acnes* and *Staphylococcus epidermidis*. These bacteria also produce sphingomyelinase, which hydrolyzes ceramides on the skin surface into free fatty acids and cholesterol. This activity further lowers skin pH and supports lipid barrier integrity, protecting against pathogenic colonization and excessive dryness [63]. Disturbances in skin pH balance, colonization by pathogens, and failure of antimicrobial defenses are strongly associated with skin diseases, including AD [21]. 

The dermis is the thickest layer of the skin, containing blood vessels, sebaceous and sweat glands, immune cells, and nerve endings. This layer ensures proper thermoregulation, participates in tactile sensation, nourishes the epidermis, and serves as an immunological barrier against microorganisms. It harbors numerous immune cells, including T lymphocytes, mast cells, tissue-resident macrophages (TRMs), and dendritic cells. Fibroblasts within the dermis play multiple crucial roles in antimicrobial defense and immune regulation. The latest advances in bioengineering demonstrate the potential of bioactive gelatin nanofibers containing lumbrokinase as a novel strategy for improving wound healing. Their mechanism of action is primarily due to their anti-inflammatory (lowering the secretion of IL-6 and TNF-α) and fibroblast-stimulating effects, and they have achieved promising results in in vitro models [64].

Fibroblasts synthesize pro-inflammatory cytokines such as IL-6, interferon-β (IFN-β), and tumor necrosis factor alpha (TNF-α), as well as chemokines like C-X-C motif chemokine ligand 1 (CXCL1) [65], and secrete growth factors and metalloproteinases that support tissue repair after infection [66]. Fibroblasts can also differentiate into preadipocytes, which produce the antimicrobial peptide cathelicidin, thereby protecting the skin against deep bacterial penetration [67]. Recent studies also highlighted the significant role of this peptide in antifungal defense against Candida albicans. Metabolic products of this fungus induce inflammatory responses through the FGFR-MEK-ERK pathway, illustrating the multifaceted contribution of dermal fibroblasts to antimicrobial defense [68]. TRMs play a critical role in skin immunity through phagocytosis, antigen presentation to naïve T lymphocytes [69], and secretion of chemokines that recruit neutrophils, including C-X-C motif chemokine receptor 2 (CXCR2) ligands [70]. Dermal T lymphocytes comprise multiple populations, including resident memory T cells, CD4^+^ helper T cells, CD8^+^ cytotoxic T cells, and γδ T cells [71]. CD4^+^ memory T cells mount rapid recall responses and secrete pro-inflammatory cytokines, such as TNF-α and IL-22, which are essential for rapid pathogen neutralization [71]. CD4^+^ helper T lymphocytes coordinate immune processes by secreting specific cytokines and chemokines that regulate inflammation and activate other immune cells, including macrophages, neutrophils, and B lymphocytes [72]. Another important component of antimicrobial defense are γδ T cells, which produce pro-inflammatory cytokines and growth factors such as fibroblast growth factor (FGF7), thereby supporting epithelial regeneration and wound healing [73]. They also exert direct cytotoxic effects through the release of perforin and granzymes, inducing apoptosis in target cells [74].

The subcutaneous tissue plays an important role in defense against pathogens. It not only serves as an insulating layer, protecting deeper organs from heat loss and mechanical injury, but also acts as a reservoir of immune cells [75]. Adipocytes within this layer secrete cytokines, adipokines, and antimicrobial peptides that contribute directly to pathogen neutralization [76].

In summary, the skin constitutes a key antimicrobial barrier, which not only provides mechanical protection according to its integrity but also serves as a reservoir of immune cells and a site of multiple immunological reactions that safeguard the body against pathogen invasion.

## 4. The Role of Vitamin D in the Pathogenesis and Treatment of Atopic Dermatitis/Immune Pathways Linking the Microbiota and Atopic Dermatitis

For more than a decade, the potential impact of VD on inflammatory processes and the severity of AD have been investigated. Through multiple regulatory mechanisms within the immune system, VD exerts beneficial effects on the course of the disease and improves patients’ quality of life [33]. The positive role of VD in AD is mediated by several mechanisms.

**Figure 7:**
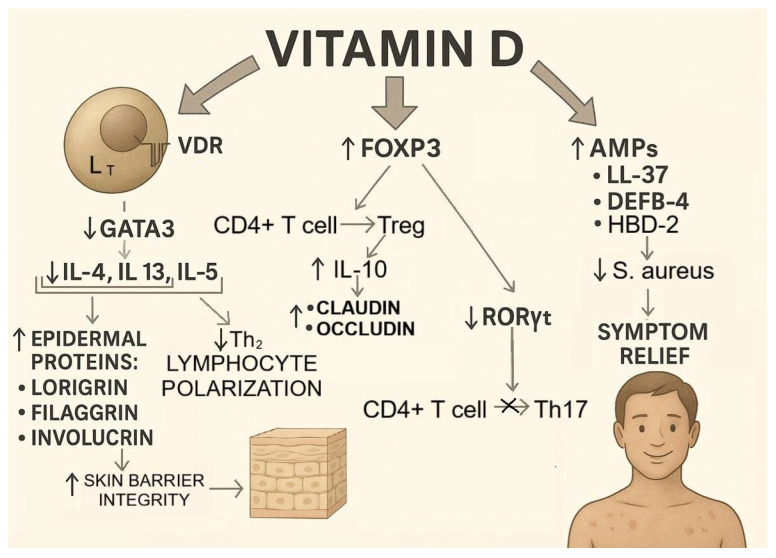
Mechanisms of Vitamin D (VD) action and their impact on atopic dermatitis (AD). VD, by binding to the VDR, inhibits GATA3 expression, leading to a decrease in IL-4, IL-5, and IL-13 production and thereby reducing Th2 cell polarization. Consequently, the reduction in IL-4 and IL-13 levels enhances the expression of epidermal structural proteins (loricrin, filaggrin, and involucrin) which strengthens the integrity of the skin barrier. Vitamin D also stimulates FOXP3 expression, promoting the differentiation of T lymphocytes into Tregs. These cells secrete IL-10, which reinforces tight junctions by upregulating the expression of claudins and occludins. At the same time, FOXP3 suppresses the transcription of the RORγt gene, resulting in reduced differentiation of Th17 cells. Furthermore, vitamin D induces the production of AMPs (LL-37, DEFB-4, and HBD-2), which inhibit the growth of pathogenic microorganisms, including *Staphylococcus aureus*, thereby alleviating the clinical symptoms of AD.

1. Inhibition of Th2 polarization: By binding to the VDR in the nucleus of T lymphocytes, VD downregulates the transcription factor GATA3 [77], which is critical for the polarization of naive CD4^+^ T cells into Th2 cells. This reduces the production of cytokines (IL-4, IL-5, and IL-13) essential for Th2 polarization, thereby preventing excessive pathological Th2 differentiation, which is a hallmark of autoimmune diseases, including AD [78].
2. Promotion of regulatory T cell (Treg) differentiation: VD enhances the expression of the transcription factor forkhead box P3 (FOXP3), the master regulator of Treg development, in naive T cells [79]. This leads to both increased Treg differentiation and suppression of Th17 differentiation, since FOXP3 inhibits the expression of RORγt, the transcription factor required for Th17 lineage commitment [80].
3. Induction of AMPs: In AD, 70–90% of lesions are colonized by *S. aureus*, which exacerbates inflammation and impairs barrier function [81]. VD increases the production of AMPs, including LL-37 [82] and human β-defensin 4 (DEFB-4) [83], which exert strong antimicrobial effects against *S. aureus*. By upregulating genes encoding AMPs, VD enhances their production and limits the pathogenic role of *S. aureus* in AD [84].
4. Upregulation of epidermal structural proteins: VD indirectly increases the expression of the key epidermal proteins filaggrin, involucrin, and loricrin by suppressing IL-4 and IL-13, which otherwise downregulate their synthesis [85]. A proper balance of these proteins ensures epidermal barrier integrity and contributes to improved AD outcomes [86] (Figure 7).

The gut microbiota is an integral modifier of AD progression through its systemic effects. Beneficial bacteria such as *Bifidobacterium*, *Roseburia*, and *Faecalibacterium* promote the production of SCFAs [87]. SCFAs exert anti-inflammatory effects in allergic and autoimmune diseases by sequentially inducing polymorphonuclear myeloid-derived suppressor cells and Tregs [88]. These findings have fueled interest in therapeutic modulation of the gut microbiota, including fecal microbiota transplantation, as a potential future intervention for AD [89]. A healthy gut microbiota also reduces AD severity by enhancing AMP production in the skin. SCFAs produced by commensals act as histone deacetylase inhibitors in keratinocytes, increasing histone acetylation at AMP gene promoters. This results in upregulated transcription of AMPs such as LL-37 and human β-defensin 2 (HBD-2) [90], leading to reduced *S. aureus* colonization and attenuated skin inflammation [91]. VD is also indispensable for maintaining both gut and skin barrier integrity. Active VD binds to VDR in intestinal epithelial cells, upregulating TJs proteins including claudins, occludins, and zonula occludens (ZO) proteins [92]. These proteins are essential for barrier function, as they prevent the translocation of pathogens and support the growth of beneficial SCFA-producing bacteria. Importantly, the gut microbiota itself modulates VDR expression. Reduced VDR expression associated with pathogenic colonization is implicated in the pathogenesis of inflammatory bowel diseases such as IBS and celiac disease [93]. Conversely, proper VDR expression in keratinocytes ensures intercellular TJs, preserving epidermal barrier integrity and limiting AD severity [94]. This highlights the crucial role of the gut microbiota not only in intestinal barrier function but also in systemic processes, including skin barrier maintenance, emphasizing the complexity of AD pathogenesis and the need for multi-targeted therapeutic strategies. Another important mechanism is the microbiota-driven regulation of cytokine balance. A healthy microbiota promotes anti-inflammatory IL-10 production via Treg induction [94]. IL-10 dampens excessive immune responses, reduces inflammation, and enhances the expression of TJ proteins such as claudins and occludins [95]. IL-17 production is also regulated by the gut microbiota. SCFAs suppress excessive IL-17 release from Th17 cells. Although physiological levels of IL-17 promote AMP synthesis [96], overproduction amplifies pro-inflammatory cytokines and chemokines, increases metalloproteinase activity (damaging the epidermis), and impairs Treg activity [97].

Another key cytokine influencing the epidermal barrier is IFN-γ, the levels of which can be reduced by SCFAs [98]. Excess IFN-γ suppresses the synthesis of barrier proteins (involucrin, filaggrin, and loricrin) [99] and inhibits ceramide production, which is essential for a functional lipid barrier [100]. IFN-γ is also a major mediator of skin inflammation in AD. The gut microbiota regulates signaling through the aryl hydrocarbon receptor (AhR), a transcription factor expressed in the gut and skin. AhR modulates detoxification, cell proliferation, and immune responses [101]. Its agonists include aromatic hydrocarbons, polychlorinated biphenyls, and dioxins, which activate detoxification pathways via cytochrome P450 genes [102]. Importantly, AhR ligands are also derived from tryptophan metabolism by gut bacteria such as *Lactobacillus*, *Bacteroides*, *Escherichia coli*, and *Clostridium*, yielding indole-3-aldehyde (IAld), indole-3-acetic acid (IAA), and kynurenine [103]. These ligands activate AhR, leading to enhanced intestinal and skin barrier integrity, increased production of epidermal structural proteins, and the promotion of anti-inflammatory Tregs [104,105]. Tolerogenic dendritic cells (tol-DCs) are another crucial component of immune modulation. These cells maintain immune tolerance and suppress inflammation [106]. SCFAs influence tol-DCs epigenetically, promoting their tolerogenic phenotype. This contributes to proper immune responses to food antigens, mitigates intestinal inflammation (e.g., in Crohn’s disease), and supports Treg induction [107]. Taken together, the interplay between gut microbiota, their metabolites, and VD highlights the importance of beneficial gut bacteria in maintaining skin health and limiting inflammation under pathological conditions. These interactions also open new therapeutic perspectives for managing inflammatory skin diseases, including AD.

## 5. Evidence from Animal Models and Mechanistic Studies

Animal models of AD provide valuable tools for precisely investigating the effects of VD on inflammation and epidermal barrier function. The classical model using a VD analog (calcipotriol/MC903) involves inducing an “AD-like” phenotype in mice (eosinophilic infiltration, type 2 immune dominance, and pruritus), mainly through the induction of TSLP (thymic stromal lymphopoietin) in keratinocytes, highlighting that excessive local VDR activation may initiate inflammation [108,109,110,111,112]. This phenomenon does not contradict the immunoregulatory role of the VD-VDR axis demonstrated in many studies, where systemic activation/supplementation of VD promotes normalization of innate and adaptive responses, in addition to supporting wound healing and epidermal homeostasis [113,114,115,116]. In an NC/Nga mouse model (spontaneous AD), topical administration of calcitriol alleviated lesion severity, reduced transepidermal water loss (TEWL), and restored tight junction integrity. Increased expression of filaggrin and loricrin, reduced IL-13/IL-33, and enhanced β-defensin expression were observed. These findings were confirmed in a Dermatophagoides farinae allergen-induced model, which provided further evidence of improvements in barrier function and inflammatory parameters [117]. 

Subsequent studies elucidated inflammatory mechanisms triggered by MC903, including activation of the TSLP → dendritic cell/Th2 axis, the involvement of IL-33, and nodal points such as mechanistic target of rapamycin complex 1 (mTORC1) and hypoxia-inducible factor-reactive oxygen species (HIF-ROS). Pharmacologic or genetic inhibition of these pathways (via rapamycin, regulatory-associated protein of mTOR knockout (Raptor-KO), or DMOG) reduced inflammation, eosinophilia, and type 2 markers [108,109,118,119].

Regarding MC903 model modulation, independent groups demonstrated that modification of innate immune co-signals, e.g., toll-like receptor 3 (TLR3) activation by poly(I:C), enhances TSLP responses and the AD-like phenotype, confirming the model’s sensitivity to environmental stimuli [120,121,122]. Although the results of human interventional trials remain heterogeneous, mechanistic studies have shown that VD supplementation increases AMP expression in the skin (LL-37), thereby improving control over *S. aureus* and other pathogens—a central element in AD pathophysiology [114,115,116,122]. VDR knockout models display impaired epidermal homeostasis (alopecia, defective keratinocyte proliferation/differentiation, and barrier dysfunction) and altered immune responses. Some VDR effects are independent of 1,25(OH)_2_D_3_, reflecting the receptor’s role as a transcription factor [123,124,125]. Reviews of studies on epithelial barrier biology further confirm that the VDR axis regulates tight junction proteins and maintains barrier integrity [92,126]. In AD patients, reduced expression of claudin-1 (CLDN1) and other TJ components correlates with higher TEWL and increased susceptibility to infections; experimental manipulation of CLDN1 in models supports its causal role in inflammation and barrier dysfunction [127,128,129].

Aquaporin-3 (AQP3), a regulator of water and glycerol transport in keratinocytes, also influences hydration and epidermal differentiation; its modulation has been linked to barrier properties and inflammatory pathways [130,131,132].

Taken together, data from animal models and ex vivo/in vitro experiments indicate that proper VD/VDR signaling

(I). strengthens barrier structure (filaggrin, loricrin, and TJ proteins);
(II). limits Th2/Th17 inflammation and alarmins (TSLP/IL-33);
(III). enhances antimicrobial defense (AMPs, especially LL-37).

In certain models, however, excessive local VDR activation (MC903) may paradoxically trigger type 2 inflammation, emphasizing the importance of dose, route of administration, and inflammatory context [108,112,114,117,133].

From a clinical perspective, reviews and meta-analyses underline the association between low 25(OH)D levels and AD severity, as well as the potential benefits of supplementation, though randomized controlled trial (RCT) results remain inconsistent, depending on population, dose, and duration [29,134,135].

In summary, a wide range of experimental models—pharmacological (MC903), spontaneous (NC/Nga), allergen-induced, VDR knockout mice, and ex vivo studies—enable a detailed investigation of VD effects on inflammation and epidermal barrier integrity. Table 1 below summarizes the key features of these models, their methods of induction, and the main observations regarding the VD/VDR axis.

## 6. Vitamin D as a Modulator of the Skin Microbiome in Atopic Dermatitis

VD is a critical modulator of the cutaneous microbiome, and VD deficiency is associated with the exacerbation of inflammatory skin diseases, including AD [136]. VD increases the diversity of the gut microbiota, limits bacterial translocation, and fosters conditions favorable for the growth of *Firmicutes*, *Bifidobacterium*, and *Lactobacillus* while constraining pro-inflammatory Proteobacteria [137]. This supports microbial diversity, intestinal barrier integrity, and the production of beneficial SCFAs. VD therefore indirectly influences the maintenance of a healthy gut and skin microbiome, leading to a reduction in the intensity of the inflammatory processes occurring in AD.

**Figure 8:**
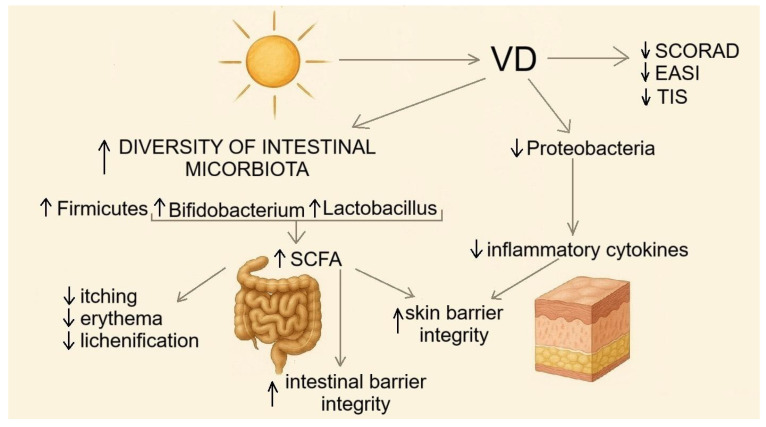
Vitamin D modulates gut microbiota composition, reduces inflammation, and enhances intestinal and skin barrier integrity. This diagram shows how vitamin D supplementation increases microbial diversity (↑ *Firmicutes*, *Bifidobacterium*, and *Lactobacillus*), promotes SCFA production, and decreases *Proteobacteria* and inflammatory cytokines. These effects improve barrier function and alleviate the clinical symptoms of atopic dermatitis (itching, erythema, and lichenification), leading to lower SCORAD, EASI, and TIS scores.

Perturbations of the gut microbiome in early childhood are an important factor in the development of inflammatory skin diseases. Breastfeeding influences the maintenance of a healthy gut microbiota: compared with infants fed formula, exclusively breastfed infants exhibit greater microbial diversity [138], higher levels of SCFA-producing bacteria (including *Bifidobacterium* and *Lactobacillus*), and lower levels of pathogenic taxa, such as *Clostridioides* and members of the Enterobacteriaceae family [139,140]. Studies also indicate that VD supports endothelial function by inhibiting the production of inflammatory cytokines [141] (Figure 8). A meta-analysis of 39 studies assessing antibiotic exposure during pregnancy and early childhood found that maternal antibiotic use increased the risk of AD in offspring by 9%, whereas antibiotic use in early childhood increased the risk by 22% [142]. These findings underscore the importance of avoiding antibiotics during pregnancy and in early life, periods of heightened vulnerability to immune-mediated diseases often linked to gut dysbiosis [143]. Collectively, these data highlight the central role of VD in the diet: VD- and probiotic-rich nutritional patterns can optimize gut microbiota composition, reduce skin inflammation, and strengthen both cutaneous and intestinal barriers, potentially mitigating allergic and autoimmune diseases [30,144]. Clinical studies further suggest therapeutic benefits of VD supplementation, reporting a moderate and statistically significant reduction in AD severity (SMD = −0.41, 95% CI: −0.67 to −0.16, *I*^2^ = 58%, *p* < 0.01) [33]. Among individuals supplementing >1000 IU/day, the effect was statistically significant, whereas lower doses did not yield significant therapeutic benefit. These observations support a potential therapeutic role of VD supplementation in AD [105].

Changes in the gut microbiome occurring under the influence of VD supplementation have a positive effect on the course of AD and help reduce the severity of persistent symptoms such as erythema, pruritus, and skin lichenification [145]. Studies indicate that individuals with AD who supplement with VD experience a reduction in symptom severity according to the SCORAD, EASI, and Three Item Severity (TIS) scales [33,146]. The complexity of the therapeutic effects of VD is illustrated in Figure 8.

## 7. Potential Therapeutic Approaches, Clinical Implications, and Future Directions

In adult patients with moderate-to-severe AD, monitoring serum 25(OH)D levels is recommended [147]. VD concentrations in these patients are typically lower than in healthy populations and correlate with disease severity [148]. Serum 25(OH)D concentration is the established marker of VD status. The most effective supplementation strategy is daily oral cholecalciferol, which remains the preferred form. Other analogs (calcifediol, calcitriol, and alfacalcidol) and parenteral administration are reserved for specific indications [149]. Currently, no universal guidelines define optimal serum 25(OH)D levels; however, most clinical studies consider 30–60 ng/mL to be adequate, <30 ng/mL to indicate deficiency, and <20 ng/mL to indicate severe deficiency [150,151]. Some evidence suggests that levels below 50 ng/mL may be insufficient [152]. Higher VD concentrations (≥50 ng/mL) appear to support optimal immune responses, and in autoimmune diseases such as psoriasis and asthma, optimal levels may reach ≥80 ng/mL, raising the possibility of similar targets for AD, though further clinical studies are needed [153].

Because dietary habits and sunlight exposure strongly influence VD metabolism, establishing universal supplementation regimens remains challenging. Individualized strategies accounting for geographic, environmental, and lifestyle factors may be required [154]. Dosing should also be adjusted for body mass index (BMI); in obesity, doses may need to be doubled or even tripled compared with standard regimens [155,156].

In a study by Borzutzky et al., children received weekly VD supplementation (8000–16,000 IU depending on age) for six weeks. This regimen did not improve SCORAD scores, symptoms, or AD biomarkers compared with placebo, although both daily and weekly dosing effectively increased serum VD levels. Symptom improvement was primarily correlated with rises in serum 25(OH)D, with the greatest benefit seen in children with severe baseline deficiency [157]. Pediatric studies administering 1000–1600 IU/day for 1–3 months reported reductions in SCORAD and EASI scores and clinical improvement [158].

According to current guidelines, the upper safe daily intake for most adults is 4000 IU, and higher doses may cause complications [159]. VD supplementation can restore epidermal integrity and enhance microbiome diversity by limiting *S. aureus* overgrowth [37]. Adverse effects are rare, and toxicity usually occurs only when serum 25(OH)D exceeds 150 ng/mL [160,161,162]. Therefore, serum monitoring is advised to individualize dosing and minimize adverse events [163]. Notably, in about half of patients, discontinuation of supplementation leads to disease relapse, suggesting that VD may act more as a preventive measure than as a therapeutic agent in preventing flares [164].

The use of calcipotriol, a VD_3_ analog, is well established in psoriasis and chronic hand eczema but has not yet been validated for AD [165,166]. Topical creams containing VD analogs at appropriate concentrations may improve lesional skin [167]. Due to their immunomodulatory and barrier-enhancing properties, these treatments remain a promising research avenue requiring further clinical trials. Although evidence is still limited, the use of topical VD analogs is a potentially valuable therapeutic option, particularly in combination with barrier-repair strategies. In adults with moderate AD, 6–8 weeks of narrowband UVB therapy increased serum VD levels, reduced EASI, SCORAD, Patient-Oriented Eczema Measure (POEM), and Dermatology Life Quality Index (DLQI) scores, and improved microbiome diversity in lesional skin [168]. These findings suggest that combining oral VD supplementation with UVB phototherapy may be a promising therapeutic strategy. Another potential direction is combining VD with probiotics, particularly *Lactobacillus* and *Bifidobacterium* strains, which may synergistically reduce inflammation and promote microbiome restoration [169,170]. Given its low cost, supplementation has high clinical value [171]. Future research should focus on identifying VDR polymorphisms, which may allow for dose optimization based on genetic variants and receptor activity [172]. CYP24A1, the gene encoding the enzyme responsible for calcitriol degradation, has clinical relevance, as increased activity may lower serum VD levels [173,174]. Elevated CYP24A1 expression has been observed in inflamed skin, suggesting that inflammation itself alters VD metabolism. This illustrates the need for studies investigating personalized supplementation tailored to dynamic metabolic pathways [31]. Low maternal VD levels during the first trimester of pregnancy are associated with an increased risk of AD in offspring [175]. Thus, supplementation during pregnancy may help prevent AD development in children [176,177]. However, evidence remains inconclusive, underscoring the need for further investigation [178]. Importantly, most studies have had short follow-up periods, emphasizing the necessity of long-term trials to evaluate both potential adverse effects and sustained clinical benefits, including reduced AD severity. Translational implications derived from current evidence on VD in AD are shown in Table 2.

## 8. Strengths and Limitations

This review comprehensively addresses the role of VD in the pathogenesis and therapy of atopic dermatitis, integrating evidence related to immune modulation, epidermal barrier function, and the gut–skin microbiome axis. The work is distinguished by its up-to-date references, clear structure, and translational perspective linking basic and clinical findings, which enhances its scientific and practical relevance.

The available studies show significant heterogeneity in patient populations, methods of assessing the microbiome, and VD status, which limits comparability across studies. There is also a lack of clear data regarding optimal dosage, duration, and the safety of long-term VD supplementation, as well as its interaction with other therapeutic modalities used in atopic dermatitis. Further clinical studies are required to standardize treatment recommendations and clarify the impact of VD supplementation on the microbiome and disease outcomes.

## 9. Conclusions

VD plays an important role in the pathogenesis of AD, acting on the immune system, the epithelial barrier, and the microbiota. VD supplementation may help alleviate AD symptoms by modulating Th2/Th17 pathways and supporting Treg activity. Evidence from clinical and animal studies shows beneficial effects on keratinocyte differentiation, antimicrobial peptide production, and strengthening of the skin barrier. Mechanistically, these effects are mediated by the VDR and downstream pathways involved in immune regulation and skin homeostasis. The interactions between VD and the microbiome suggest a potential role in restoring immune and barrier homeostasis in AD. In the gut microbiota, SCFA-dependent mechanisms and activation of the AhR appear to be critical, while in the skin microbiome, processes are primarily driven by increased production of AMPs and stabilization of the lipid barrier. Despite promising results, optimal dosing, treatment duration, and combination approaches remain insufficiently defined. Further large-scale clinical and experimental studies are needed to confirm the efficacy of VD supplementation and develop standardized clinical guidelines.

## Figures and Tables

**Table 1:** Animal and cellular models of AD used to investigate the role of VD/VDR in inflammation and epidermal barrier function.

Model	Experimental Conditions	AD-like Phenotype/Main Readouts	Effect of VD/VDR
Ex vivo/in vitro [92,116]	Human keratinocytes/skin; stimulation with 1,25(OH)_2_D_3_	Analysis of filaggrin, loricrin, claudin-1, AMP (LL-37)	VD/VDR strengthens barrier, AMP expression, and limits alarmins
MC903 (calcipotriol) [120,121]	C57BL/6 mice; topical application of MC903 (0.05–0.1 µg/day for 5–10 days)	Eosinophilic infiltration, Th2 inflammation, pruritus, ↑TSLP/IL-33	Excessive local VDR activation → induction of TSLP and inflammation
NC/Nga (spontaneous AD) [117]	NC/Nga mice; topical calcitriol (1–2 µg/g/day, 2–3 weeks)	Chronic lesions, ↑TEWL, barrier defects, Th2	Calcitriol ↓TEWL, ↑filaggrin/loricrin, ↓IL-13/IL-33, ↑β-defensins
Allergen model (Dermatophagoides farinae) [117]	BALB/c mice; sensitization + challenge with mite allergen; topical calcitriol	Th2 response, barrier dysfunction, pruritus	Calcitriol reduces inflammation and improves barrier function
MC903 + poly(I:C) [120]	As above, + poly(I:C) (TLR3 agonist) topically	Exacerbation of inflammation and pruritus, ↑TSLP	Model sensitive to environmental/infectious factors
VDR knockout [123,125]	VDR-/- mice (various genetic backgrounds)	Alopecia, severe barrier defects, impaired keratinocyte differentiation	Demonstrates importance of VDR independent of 1,25(OH)_2_D_3_

Note: Arrows indicate direction of change (↑ increase, ↓ decrease).

**Table 2:** Translational implications derived from current evidence on Vitamin D in atopic dermatitis.

Area	Key Insights	Clinical Implications	Future Directions
Serum 25(OH)D monitoring	Lower VD levels correlate with AD severity [148]	Routine testing recommended; most studies define <30 ng/mL as deficient [150,151]	Determine optimal therapeutic thresholds (e.g., ≥50–80 ng/mL) [152,153]
Oral supplementation	Daily cholecalciferol most effective [149]	Individualized dosing; obesity requires higher intake [154,155,156]	Establish optimal dose and duration; long-term trials needed [153]
Topical VD analogs	Calcipotriol validated in psoriasis; limited AD data [165,166,167]	May improve barrier function and reduce inflammation [166,167]	Conduct controlled studies to confirm efficacy and safety [165,166,167]
Phototherapy (NB-UVB)	NB-UVB increases VD levels and improves clinical scores [168]	Effective adjunctive therapy for moderate AD [168]	Assess combination strategies (UVB + oral VD)
Microbiome modulation	VD reduces *S. aureus* overgrowth and increases diversity [36]	Supports microbiome restoration and barrier repair [36]	Explore mechanistic pathways affecting skin microbiome
Probiotics	*Lactobacillus*/*Bifidobacterium* synergize with VD [169,170]	Combined supplementation may reduce inflammation [169]	Clarify which strains and doses are optimal
Genetic factors (VDR, CYP24A1)	Polymorphisms influence VD metabolism and response [172,173,174]	Potential for personalized dosing [172]	Pharmacogenomic profiling recommended
Pregnancy and early development	Low maternal VD linked with AD risk [168]	Supplementation in early pregnancy may reduce risk [169,170]	Further studies needed due to conflicting evidence [171]
Safety	Adverse effects are rare; toxicity >150 ng/mL [153,154,155]	Monitoring recommended, especially with higher doses [156]	Studies on long-term safety required
Relapse after discontinuation	Flares may return when VD is stopped [157]	Consider continuous maintenance supplementation [157]	Compare continuous vs. intermittent treatment regimens

## Data Availability

We used PubMed, ScienceDirect, and Google Scholar databases to screen articles for this review. We did not report any data.

## Abbreviations

The following abbreviations are used in this manuscript:

1,25(OH)_2_D_3_	1,25-Dihydroxycholecalciferol, calcitriol
1,25(OH)_2_D_2_	1,25-Dihydroxyergocalciferol, ercalcitriol
25(OH)D	25-Hydroxyvitamin D
7-DHC	7-dehydrocholesterol
1,25(OH)2D	1,25-Dihydroxyvitamin D
AD	Atopic dermatitis
AhR	Aryl hydrocarbon receptor
AMPs	Antimicrobial peptides
AQP3	Aquaporin 3
BMI	Body mass index
CD	Cluster of differentiation
CLDN1	Claudin-1
CXCL1	C-X-C motif chemokine ligand 1
CXCR2	C-X-C motif chemokine receptor 2
DBP	Vitamin D-binding protein
DEFB-4	Human β-defensin 4
DLQI	Dermatology Life Quality Index
DMOG	Dimethyloxalylglycine
EASI	Eczema Area and Severity Index
FGF7	Fibroblast growth factor 7
FLG	Filaggrin
FOXP3	Forkhead box P3
HBD-2	Human β-defensin 2
HIF-ROS	Hypoxia-inducible factor—reactive oxygen species
IAld	Indole-3-aldehyde
IAA	Indole-3-acetic acid
IFN	Interferon
Ig	Immunoglobulin
IL	Interleukin
JAK1	Janus kinase 1
LL-37	Human cathelicidin antimicrobial peptide
MC903 (Calcipotriol)	Vitamin D analog used experimentally in dermatology
mTORC1	Mechanistic target of rapamycin complex 1
NPC1L1	Niemann-Pick C1-like protein 1
POEM	Patient-Oriented Eczema Measure
RCT	Randomized controlled trial
Raptor-KO	Regulatory-associated protein of mTOR knockout
SCFA	Short-chain fatty acids
SCORAD	Scoring Atopic Dermatitis
SR-BI	Scavenger receptor class B type I
STAT6	Signal transducer and activator of transcription 6
TEWL	Transepidermal water loss
Th	T helper cells
TIS	Three-Item Severity
TJs	Tight junctions
TLR3	Toll-like receptor 3
tol-DCs	Tolerogenic dendritic cells
TNF-α	Tumor necrosis factor alpha
TRMs	Tissue-resident macrophages
Treg	Regulatory T cells
TSLP	Thymic stromal lymphopoietin
UVB	Ultraviolet B radiation
VD	Vitamin D
VD2	Vitamin D2 (ergocalciferol)
VD3	Vitamin D3 (cholecalciferol)
VDR	Vitamin D receptor
ZO	Zonula occludens
